# Heparin modulation on hepatic nitric oxide synthase in experimental steatohepatitis

**DOI:** 10.3892/etm.2014.1963

**Published:** 2014-09-15

**Authors:** AMAL HASSANIN, HALA ABDEL MALEK, DALIA SALEH

**Affiliations:** 1 Department of Clinical Pharmacology, Faculty of Medicine, Mansoura University, Mansoura 35516, Egypt; 2 Department of Anatomy and Embryology, Faculty of Medicine, Mansoura University, Mansoura 35516, Egypt

**Keywords:** heparin, fatty liver, inducible nitric oxide synthase, mice

## Abstract

Nonalcoholic fatty liver disease (NAFLD) is considered to be a hepatic manifestation of metabolic syndrome, and has been etiologically associated with insulin resistance (IR). The histopathology of NAFLD ranges between simple steatosis and nonalcoholic steatohepatitis (NASH), with or without fibrosis. The aim of the present study was to examine the effect of heparin on steatohepatitis and hepatic-induced nitric oxide synthase (iNOS) expression in mice. Male mice were divided into four groups, which included the normal basal diet (control), high fat (HF) diet, HF diet + heparin (treatment group) and heparin control groups. After eight weeks from the initiation of the experiment, blood was collected and livers were harvested for biochemical analysis and histological studies. Serum levels of aspartate aminotransferase, alanine aminotransferase, hepatic triglyceride (TG) and hydroxyproline, as well as the IR, superoxide anion generation and mRNA expression of the hepatic iNOS enzyme were evaluated. Liver specimens were processed for histopathological and immunohistopathological evaluation. Heparin administration decreased the levels of the liver enzymes, IR, superoxide generation, hepatic TG, hydroxyproline and iNOS expression when compared with the HF diet group. These changes were associated with an improvement in inflammation and fibrosis observed via histopathological examination. Therefore, heparin treatment attenuates hepatic injury in steatohepatitis.

## Introduction

Nonalcoholic fatty liver disease (NAFLD) occurs in individuals that have not consumed alcohol in amounts considered harmful to the liver. The disease is characterized by macrovesicular hepatic steatosis. There are two histological patterns of NAFLD: Fatty liver alone and steatohepatitis. Nonalcoholic steatohepatitis (NASH) is defined as lipid accumulation in the liver with evidence of cellular damage, inflammation and varying degrees of scarring or fibrosis (1).

The mechanisms underlying the increase in hepatic fat accumulation are unclear and little information is available on the time course of the development of hepatic steatosis. The association between macrovesicular steatosis of the liver and inflammatory changes and fibrosis in obese individuals has been reported for several decades (2). The spectrum of this condition ranges between silent nonalcoholic fatty liver disease and chronic liver disease with complications. Animal models indicate that the liver may accumulate lipids within a few weeks or even a few days (3).

NAFLD is present in the majority of patients with metabolic risk factors, including obesity and diabetes. Insulin resistance (IR) is a key pathogenic factor of hepatic fat accumulation. NAFLD exacerbates hepatic IR and precedes glucose intolerance. Once hepatic steatosis occurs, additional factors, including oxidative stress, mitochondrial dysfunction and adipocytokines, may enhance hepatocellular damage (4).

NASH may progress to cirrhosis, particularly when features of bridging fibrosis are present. No definite therapy is currently available to reverse this pathological process (4). A number of studies have investigated the efficacy of medications for the treatment of NASH; the drugs studied include orlistat, metformin, vitamin E, lipid-lowering drugs, pentoxifylline and pioglitazone (4,5). In addition, thrombin inhibitors have been considered as a treatment for liver fibrosis (6), and have been found to decrease the lung collagen involved in pulmonary fibrosis (7–8).

The current study aimed to examine the effects of heparin, a thrombin inhibitor, on hepatic injury in an experimental model of NASH and IR, via analyzing the effects on hepatic inducible nitric oxide synthase (iNOS) expression and superoxide generation.

## Materials and methods

### Drugs

Heparin sodium (Nile Pharma, Cairo, Egypt) was administered subcutaneously to the mice in the herparin groups at a dose of 10 μg/day (8).

### Animals

A total of 80 male BALB/c mice (weight, 20–23 g; age, 5–7 weeks) were obtained from the Animal Center of the Research Unit at the Faculty of Medicine of Mansoura University (Mansoura, Egypt). The experiment was performed in accordance with the Guide for the Care and Use of Laboratory Animals, and the experimental procedures were approved by the local Animal Care and Use Committee of Mansoura University (Mansoura, Egypt). The animals were housed in plastic cages with a 12-h light/dark cycle, with free access to food and water throughout the experimental period.

### Experimental design

Following acclimatization for one week through consumption of a basal diet, the mice were divided into four groups (n=20) and fed the following diets for eight weeks. Control group mice were fed a normal basal diet. The mice in the high fat (HF) diet group were fed a diet containing 71% energy from fat, 11% energy from carbohydrates and 18% energy from proteins (9). Mice in the HF diet + heparin-treated group were administered 10 μg heparin sodium via daily subcutaneous injections, following the initial consumption of the HF diet for one week (second week overall) and for the remaining weeks (6–8). Finally, mice in the heparin control group were fed the normal basal diet, but administered heparin as previously described.

### Specimen collection and staining

Eight weeks from the start of the experiment, mice were anesthetized with an intraperitoneal injection of sodium pentobarbital (50 mg/kg body weight). Blood samples were collected for biochemical analysis and the livers were harvested for iNOS gene expression, histological and immunohistochemical examinations.

### Biochemical analysis

Serum levels of aspartate aminotransferase (AST) and alanine aminotransferase (ALT) were measured using an automated technique (Clinical Chemistry Analyzer), while serum glucose levels were measured using a OneTouch^®^ Ultra^®^ Blood Glucose meter (LifeScan, Inc., Milpitas, CA, USA). In addition, serum insulin levels were measured with an enzyme-linked immunosorbent assay (ELISA) kit (Crystal Chem, Inc., Downers Grove, Chicago, IL, USA), according to the manufacturer’s instructions. The level of hepatic triglyceride (TG) was measured using an enzymatic method (Sigma-Aldrich, St. Louis, MO, USA), according to the manufacturers’ instructions (10), while the hepatic hydroxyproline content was estimated, as described in a study by Bergman and Loxley (11). Hepatic superoxide anion generation was also estimated using a previously described method (12). The gene expression levels of iNOS were determined in the livers of the aforementioned groups by semi-quantitative polymerase chain reaction (PCR). In addition, PCR was performed with the livers of a fifth group of mice (n=12) that received high fat diet and subcutaneous injections of a standard nonselective iNOS inhibitor, NG-monomethyl-L-arginine (L-NMMA; Sigma-Aldrich), at a dose of 25 mg/kg/day (as with the administration of the heparin regimen) (13) to inhibit the iNOS synthase enzyme.

Total RNA was extracted from tissue specimens using a column technique, according to the manufacturer’s instructions (Vivantis Technologies, Subang Jaya, Malaysia). In total, 1 μg total RNA was reverse transcribed to cDNA using a Maxima First Strand cDNA Synthesis kit (Thermo Fisher Scientific, Ontario, Canada). A total of 2 μl cDNA was amplified in a thermal cycler instrument (Arktik Thermal Cycler; Thermo Fisher Scientific, Pittsburgh, PA, USA) with 25 pmol each primer pair, 10 mM of Tris-HCl, pH 8.3, 50 mM of KCl, 1.5 mM of MgCl_2_, 0.3 mM of dNTP, 12.5 μl 2X *Taq* PCR Master mix (Qiagen, Inc., Valencia, CA, USA) and nuclease-free water in a total volume of 25 μl.

The primers designed for iNOS were 5′-CTCGGA ACTGTAGCACAGCA3′ (sense) and 3′-GCACATCAAAGC GGCCATAG5′ (antisense). β-actin was included as a reference gene, and had primer sequences of 5′-CAGGATTCCATA CCCAAGAAG-3′ (sense) and 3′-AACCCTAAGGCCAAC CGTG-5′ (antisense). The cycling parameters of the PCR amplification were as follows: Initial denaturation at 95°C for 5 min, followed by 35 cycles of denaturation at 94°C for 30 sec, annealing at 60°C for 30 sec and extension at 72°C for 45 sec, which was followed by a final extension at 72°C for 5 min. PCR products were electrophoresed in 2% agarose gel (Vivantis Technologies) and visualized with an ethidium bromide stain (Sigma-Aldrich). A 100-bp DNA ladder (Vivantis Technologies) was applied to the first well to identify the molecular weight of the samples. Gel images were captured and analyzed with a Gel Documentation system (Bio-Rad, Hercules, CA, USA).

### Histological procedure

Small pieces of liver were preserved in 10% formal saline for ≥48 h, dehydrated in ascending grades of alcohol, cleared in xylene, embedded in paraffin and sectioned into 5-μm thick sections. The sections were stained with hematoxylin and eosin to detect the histopathological changes, while Masson’s trichrome and Sirius red stains were used to detect collagen. An α-smooth muscle actin (α-SMA; Sigma-Aldrich, St.Louis, MO, USA) immunohistochemical stain was used to detect activated Ito cells.

### Immunohistochemical procedure

A three-step indirect immunohistochemical technique was performed on 5-μm formalin-fixed and paraffin-embedded sections. Antigen retrieval was achieved by heating the sections in a microwave oven at 560 W for 21 min in citrate buffer (pH 6.0). The sections were subsequently treated with methanol containing 0.3% hydrogen peroxide for 15 min at room temperature in order to inactivate the endogenous peroxidase. Nonspecific binding of the secondary antibodies was minimized by incubation with 50% normal goat serum in phosphate-buffered saline (PBS) for 20 min. Sections were incubated with appropriate primary antibodies (α-SMA 1A4; 1/50 dilution) and diluted in PBS for 1 h in a humid chamber at room temperature. All rinsing procedures and serum dilutions were performed in PBS (pH 7.2–7.4). The detection kit used was a Dako Cytomation LSAB^™^2 System-HRP for rabbit and mouse (K0675; Dako Cytomation, Glostrup, Denmark). Positive reactions were visualized by applying DAB+ liquid (K3468; Dako, Carpinteria, CA, USA) for 5–10 min. Counterstaining with hematoxylin was conducted for 2 sec and Aqueous Glycergel^®^ Mounting medium (C563; Dako) was used to mount the stained sections.

### Statistical analysis

Data are expressed as the mean ± standard deviation. Analysis of variance followed by Tukey’s post-hoc test was used to analyze the data, where P≤0.05 was considered to indicate a statistically significant difference. All analyses were conducted using SPSS 11.0 software for Windows (SPSS, Inc., Chicago, IL, USA).

## Results

### Effects of heparin injection on serum levels of ALT and AST

When compared with the control group, the serum levels of ALT and AST were significantly higher in the HF diet group (P≤0.05). The increment of these parameters was significantly attenuated in the HF + heparin group (P≤0.05; Table I) when compared with the HF diet group.

### Effects of heparin injection on oxidative stress, hepatic TG and hepatic fibrosis

As shown in Table I, the levels of superoxide free radicals, hepatic hydroxyproline, TGs and serum insulin were significantly higher in the HF diet group when compared with the control group (P≤0.05). This increase was significantly reduced in the heparin-treated groups (HF + heparin only; P≤0.05) when compared with the HF diet group.

### Effect of heparin on the mRNA expression of iNOS

**Figure 1:**
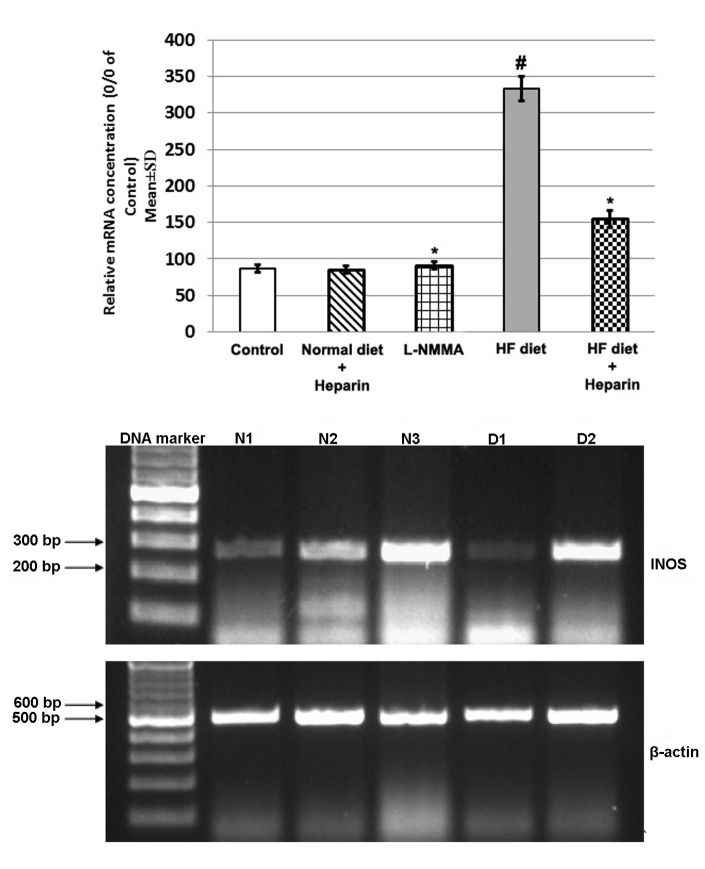
Effect of heparin administration on the mRNA expression levels of inducible nitric oxide synthase. Data are expressed as the mean ± standard deviation (n=12 per group), and presented as the percentage of the control values following comparison by analysis of variance and Tukey’s test. ^#^P≤0.05 was considered to indicate a statistically significant difference in comparison to the control group. ^*^P≤0.05 was considered to indicate a statistically significant difference in comparison to the HF diet group. N1, control group; N2, control heparin group; N3, L-NMMA-treated high fat (HF) diet group; D1, HF diet (model) group; D2, heparin + HF diet group. L-NMMA, NG-monomethyl-L-arginine.

Hepatic expression of iNOS was higher in the HF diet group compared with the control group. However, heparin treatment decreased iNOS expression when compared with the L-NMMA-treated HF diet group (Fig. 1).

### Histopathological observations of NAFLD

**Figure 2:**
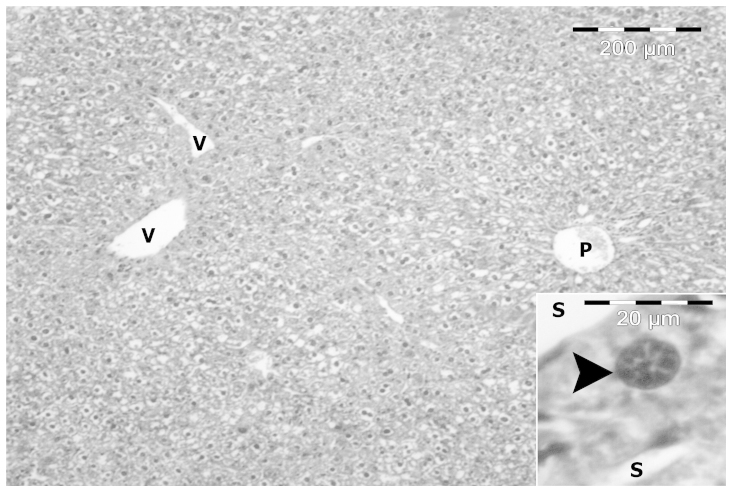
A photomicrograph (magnification, ×40; hematoxylin and eosin stain) of the liver section of a control mouse showing one portal triad (P) and two central veins (V) surrounded by hepatic cords and sinusoids. Inset: High magnification image (x400) of the hepatic cords showing a large polygonal hepatocyte with a vesicular nucleus (arrowhead) and blood sinusoids (S).

In the control group, livers were composed of a number of lobules consisting of thin-walled central veins from which cords of hepatocytes radiated towards the lobule periphery and alternated with narrow irregular blood sinusoids. Around the periphery of each lobule, branches of the hepatic artery, hepatic portal vein and bile duct were present forming the portal tract. The hepatocytes were polygonal with a large vesicular nucleus (Fig. 2).

**Figure 3:**
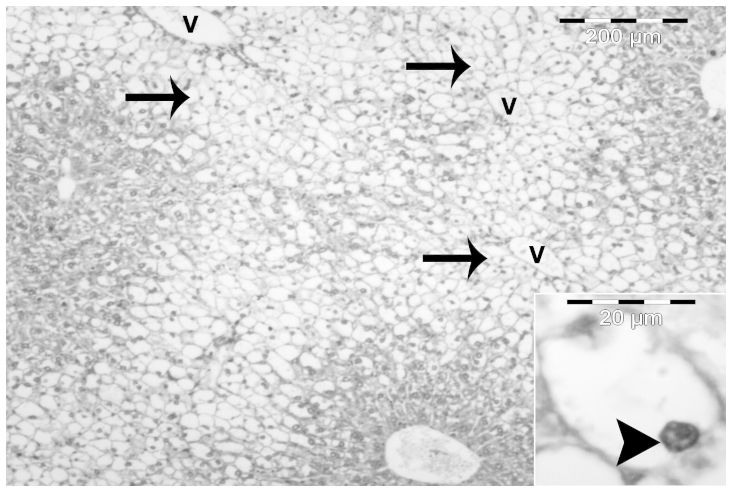
A photomicrograph (magnification, ×40; hematoxylin and eosin stain) of the liver section of a high fat-diet fed mouse showing nonalcoholic fatty liver disease in the form of severe macrovesicular (arrows) steatosis, which is present mainly in the perivenular region (V) or acinar zone 3. Inset: High magnification image (x400) showing a distended hepatocyte with a signet ring appearance (arrowhead). The cytoplasm is filled with a large empty vacuole and the nucleus is pushed to the periphery.

**Figure 4:**
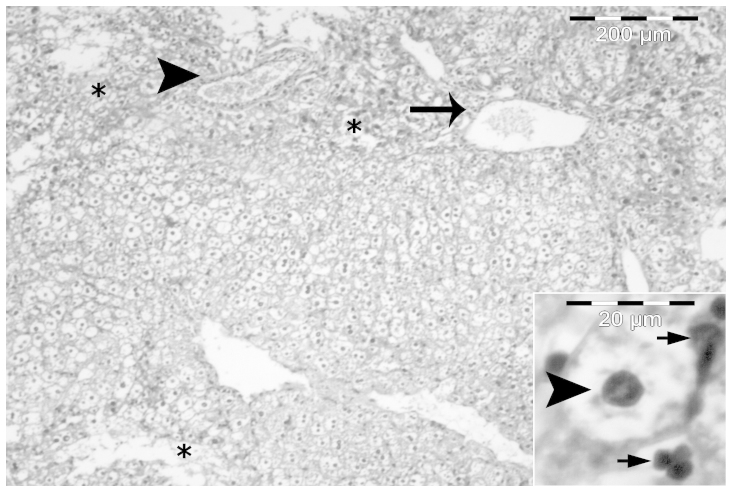
A photomicrograph (magnification, ×40; hematoxylin and eosin stain) of the liver section of a high fat-diet fed mouse showing nonalcoholic steatohepatitis in the form of mild periportal (arrow) and perivenular (arrowhead) inflammatory cell aggregation with panacinar macrovesicular steatosis. Areas of liver necrosis and destructed liver architecture are marked with an ‘^*^’. Inset: High magnification image (x400) showing the ballooning of a hepatocyte with rarefied cytoplasm (arrowhead) and inflammatory cells (small arrows) nearby.

The most important histological characteristic of the HF diet group was the presence of severe macrovesicular and microvesicular steatosis in the hepatocytes. The steatosis was predominantly centrilobular and macrovesicular (Fig. 3). However, in certain sections, steatosis was observed throughout the lobule. Macrovesicular steatosis was characterized by large vacuoles occupying almost the entire cytoplasm and forcing the nucleus to the periphery of the cell. By contrast, microvesicular steatosis was characterized by multiple small lipid vacuoles with the nucleus located in the center of the cell (Figs. 3 and 4).

**Figure 5:**
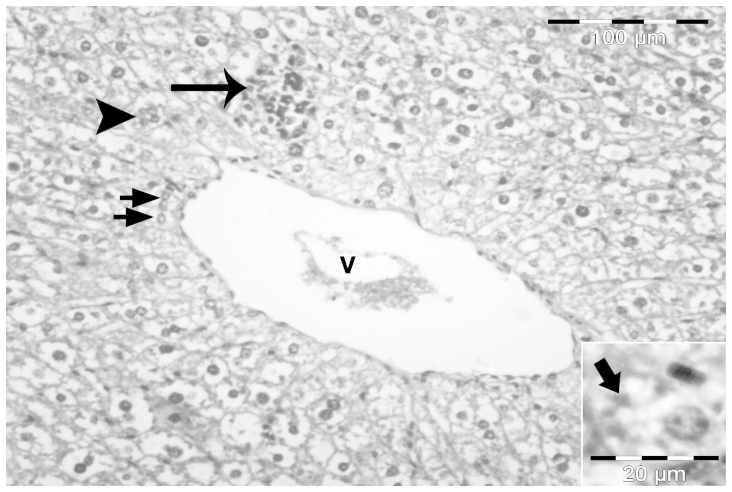
A photomicrograph (magnification, ×40; hematoxylin and eosin stain) of the liver section of a high fat-diet fed mouse showing nonalcoholic steatohepatitis in the form of focal necrosis with inflammatory cell aggregation (arrow) close to a dilated central vein (V). The lipogranuloma (arrowhead) and cells with glycogenated nuclei (short arrows) are marked. Inset: High magnification image (x400) showing a hepatocyte with Mallory’s hyaline bodies in the form of eosinophilic and amorphous structures in the cytoplasm (thick arrow).

**Figure 6:**
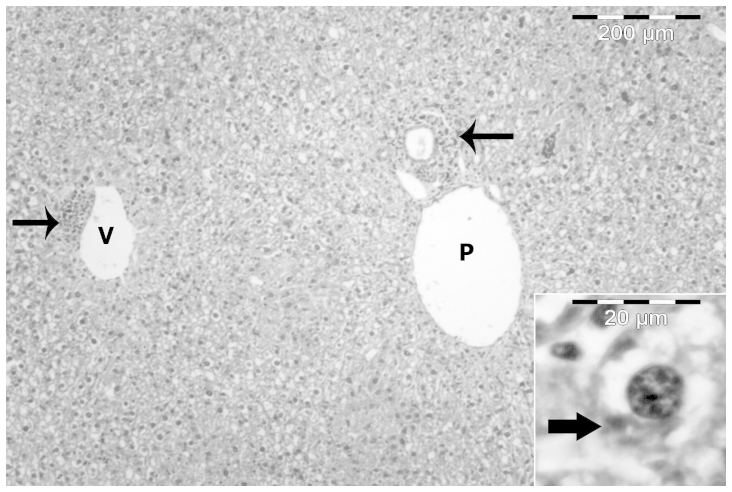
A photomicrograph (magnification, ×40; hematoxylin and eosin stain) of the liver section of a high fat-diet + heparin-treated mouse showing preserved liver architecture and a decrease in steatosis. Inflammatory cell aggregation (arrows) was observed around the portal area (P) and the central vein (V). Inset: High magnification image (x400) showing a hepatocyte with Mallory’s hyaline bodies in the form of eosinophilic and amorphous structures in the cytoplasm (thick arrow).

A number of animals developed NASH, where steatosis was accompanied by mild intralobular and periportal inflammation with prominent hepatocellular ballooning (Fig. 4). Certain sections also demonstrated fat cysts and lipogranulomas, glycogenated nuclei and cells containing Mallory’s hyaline (Fig. 5). In the heparin-treated group, steatosis and necroinflammatory activity was minimal (Fig. 6).

### Heparin treatment improved liver fibrosis

**Figure 7:**
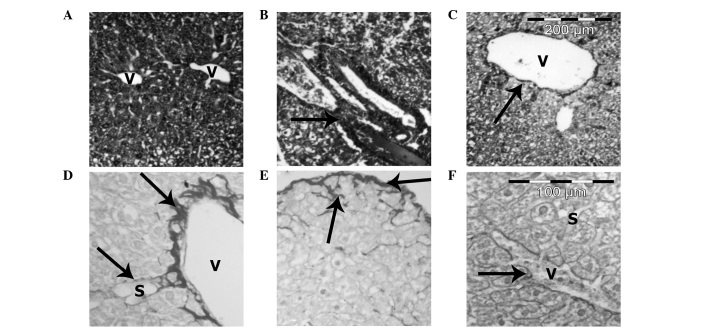
Photomicrographs of the mouse liver sections. (A) Normal control mouse liver showing normal arrangement of the central vein (V) and an intact parenchyma. (B) Liver section of high fat (HF) diet fed mice showing increased periportal fibrosis and development of a large septa (arrow) of connective tissue penetrating into the parenchyma. (C) Liver section of HF diet + heparin treated mice showing the central vein (V) with a decrease in perivenular fibrosis (arrow). (D) Liver section of high fat (HF) diet fed mice showing increased fibrosis (arrows) around the central vein (V) and blood sinusoids (S), (E) Liver section of high fat (HF) diet fed mice showing thickening of the liver capsule (arrows with a spread of fibrous tissue around the hepatocytes), and (F) Liver section of HF diet + heparin treated mice showing progressive breakdown of the fibrous septa (arrows) around the sinusoids (S) and the central vein (V). (A,B,C) Masson’s trichrome stain; magnification, ×40. (D,E,F) Siruis red stain; magnification, ×100.

Masson’s trichrome and Sirius red stains for collagen revealed minimal fibrosis in the control group (Fig. 7A). However, the extent of fibrosis increased in the HF diet group, particularly in the perisinusoidal area in zone 3 and in the periportal region. A number of animals also demonstrated bridging fibrosis and pericellular fibrosis (Fig. 7B, D and E). However, heparin treatment decreased the extent of fibrosis when compared with the HF diet group (Fig. 7C and F).

### Immunohistochemistry

**Figure 8:**
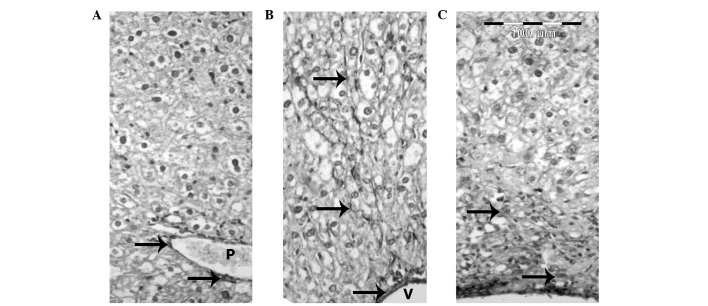
Photomicrographs (magnification, ×100) of mouse liver sections using an α-smooth muscle actin (SMA) immunoperoxidase stain. (A) Normal control mouse liver section showing α-SMA positive cells (arrows) around the portal vein (P). (B) High fat (HF)-diet mouse liver section showing increased α-SMA positive cells (arrows) in the wall of the central vein (V) and the perisinusoidal area. (C) HF-diet + heparin-treated mouse liver section showing few positive α-SMA cells (arrows).

In the liver sections of the control mice, a positive reaction between the smooth muscle cells of the terminal and sublobular venous blood vessels was observed (Fig. 8A). In the HF diet group, Ito cells reacted positively with the α-SMA antibody in the areas of fibrosis and in the walls of the central veins, as well as in the septal connective tissue (Fig. 8B). The livers of mice from the HF diet + heparin-treated group revealed fewer α-SMA positive cells compared with the HF diet group (Fig. 8C).

## Discussion

NAFLD is present in the majority of patients with metabolic and/or multiple risk factors, including obesity, diabetes and hypertension. There are two histological patterns of NAFLD: Fatty liver and steatohepatitis. NASH is defined as lipid accumulation associated with cell damage, inflammatory changes and fibrosis (1). NASH is a serious problem, with almost 25% of cases progressing to cirrhosis and subsequent complications of portal hypertension, liver failure and hepatocellular carcinoma (14).

In the present study, HF diet-fed mice were used to examine the effect of heparin on iNOS enzyme expression. The HF diet induced a pathology similar to human NASH, including steatosis, hepatic inflammation and fibrous tissue formation. Mice fed a HF diet for eight weeks also developed hyperglycemia and hyperinsulinemia, which mimicked the human metabolic syndrome process. These experimental protocols were selected in accordance with the study by Lieber *et al* (9), who reported that rats fed a HF diet *ad libitum* for three weeks demonstrated IR, as well as marked panlobular steatosis, increased hepatic lipid concentrations and oxidative damage.

The pathogenesis of steatohepatitis caused by metabolic factors is not fully understood; however, the induction of fat into the liver is essential. In experimental steatohepatitis caused by a lipogenic diet, hepatic steatosis represents the hepatic part of the metabolic syndrome disorder and is accompanied by IR and elevated TGs (2,15).

The mechanism responsible for the increase in hepatic lipid accumulation is uncertain and little information is available regarding the time course of the development of hepatic steatosis. It has been hypothesized that fatty liver may result from increased delivery of free fatty acids (FFAs), impaired hepatic fatty acid oxidation and/or impaired synthesis or secretion of very low-density lipoproteins (16). Among these factors, the increased flow of FFAs to the liver is considered the most important (17). An increased rate of lipolysis and FFA abundance are the causes of oxidative stress in the liver. FFA oxidation generates oxidative stress and subsequent lipid peroxidation, which may be the mediator of subsequent inflammatory processes (18).

Oxidative stress is a mechanism of hepatocellular injury in steatohepatitis and is associated with the accumulation of lipid peroxidation products, elevation of proinflammatory cytokines and mitochondrial dysfunction (18–19). However, excess hepatic fat may lead to hepatic IR via the release of cytokines from Kupffer cells, leading to hepatocellular inflammation, reduced endothelial nitric oxide (eNO) signaling and the development of IR, which is a risk factor for liver fibrosis in steatohepatitis (20).

Excessive circulating FFAs exert deleterious effects on mitochondria and insulin signaling via protein kinase C activation and the inhibition of insulin receptor tyrosine kinase activity, leading to systemic IR (21).

The hepatic fibrosis observations in the present study, which resulted from TG accumulation and steatohepatitis, were consistent with those reported in the study by Poniachik *et al* (22), who concluded that the degree of steatosis in the liver biopsy is a risk factor for the development of fibrosis. In addition, Larter *et al* (23) revealed that in a nutritional model of steatohepatitis, accumulation of TGs occurs despite substantial suppression of lipogenesis and the induction of TG synthesis genes; these observations support the lipotoxicity mechanism involved in the liver injury component of metabolic syndrome.

Furthermore, the origin of the reactive oxygen species increase in NASH has been investigated. Reduced superoxide scavenging and glutathione replenishment may be involved in steatohepatitis, and antioxidant therapy may have a beneficial therapeutic effect for this purpose (24–25).

Administration of a HF diet in mice induces the proinflammatory activation of Kupffer cells, which is associated with reduced liver endothelial NOS activity. Kupffer cells produce the free radical, NO, from iNOS when they are stimulated by inflammatory cytokines or lipopolysaccharide (13–26).

Liu and Huang (27) supported the association between obesity-induced IR and the reduction in the eNO vascular content that causes a predisposition to increased endothelial inflammation, thrombosis and vasoconstriction. Furthermore, Tateya *et al* (28) revealed that a HF diet induces liver inflammation and IR. The authors determined that a reduction in eNO signaling leads to increased inflammation, which is inhibited by increasing eNOS activity.

Immunohistochemical examination of the livers of the mice in the HF diet group demonstrated increased expression levels of α-SMA in the perisinusoidal stellate cells in areas of fibrosis around the portal spaces and fibrous septa. These results have been reported during the course of the development of hepatic fibrosis in humans and rats (29–32). Activated hepatic stellate cells synthesize tissue inhibitors of matrix metalloproteinase 1 and 2, which inhibit interstitial collagenase activity and contribute to the accumulation of extracellular matrix proteins; the latter of which is involved in the progression of chronic hepatic fibrosis (33–36).

NASH therapy remains under investigation and no definite therapy is currently available to reverse the cirrhotic pathological process (2). Previously, heparin was used in the management of vascular thrombosis. Heparin is a highly sulfated polysaccharide isolated from mast cells, which has been described as having antioxidant and antifibrotic activities. The drug has been used in the treatment of hepatic cholestasis and fibrosis (6,7,37). In the present study, subcutaneous injections of heparin into mice fed a HF diet for eight weeks was shown to improve liver enzyme function, IR and hepatic fibrosis via the prevention of hepatic inflammation, improved IR and decreased signaling of iNOS. Heparin is known to have biological effects that are not associated with anticoagulant activity, including potential anti-inflammatory, immunomodulatory and antitumor effects. Certain studies have demonstrated that these actions may be hindered by the effect of heparin on hemostasis; however, not in the dosage used in the present study (6,8,37).

The clinical benefit of heparin on chronic inflammatory disease has been previously studied. Heparin controls inflammation through trapping and neutralizing the inflammatory pathological proteins involved in the inflammatory process. This ability may be the result of its large polysaccharide chemical structure that binds to the different inflammatory mediators and cytokines, including antithrombin, heparin binding proteins and adhesion molecules (37). Mu *et al* concluded that heparin ameliorates lung injury and causes pulmonary vascular remodeling via the inhibition of iNOS expression (38–39). Furthermore, Ding *et al* (40) revealed that unfractionated heparin reduces inflammation and cytokine expression in endotoxemic mice. In addition, Saï *et al* (8) demonstrated that heparin attenuates low-dose streptozotocin-induced immune diabetes in mice in a dose similar to the dose used in the present study, but for a period of almost three weeks, as well as inhibiting the β-cell binding of T-splenocytes *in vitro*. The authors hypothesized that heparin at this dosage exhibits a variety of functions beyond an anticoagulant effect, without inducing the side effect of blood loss.

Tokuyama *et al* (41) revealed that diabetic patients who were administered insulin lispro and heparin treatment achieved improved glycemic control without any adverse effects. In addition, Shah and Shah demonstrated that heparin preserves hepatic function and reduces the severity of hepatic fibrogenesis following six weeks of treatment. Thus, heparin inhibits thrombin *in vivo* and *in vitro*. Thrombin is responsible for stimulating the proliferation of hepatic stellate cells (6). It interacts with specific cellular upregulated receptors that are located not only in the liver, but also in the lungs, resulting in the accumulation of collagen and fibrosis. Heparin is involved in the inhibition of the coagulation system and prevents acute liver injury from a hepatotoxic dose of lipopolysaccharide (6,42).

In conclusion, heparin administration improves the liver histopathology of mice receiving a HF diet. This effect occurs via the downregulation of the iNOS gene and the suppression of hepatic TG content. The anti-inflammatory mechanism of heparin should be determined in future studies.

## Figures and Tables

**Table I:** Effect of heparin on various parameters in a NASH mouse model.

Parameter	Control	HF diet	HF + heparin	Heparin control
Serum insulin (μU/ml)	10.83±1.37	33.33±3.97^a^	12.33±1.73^b^	11.67±2.40^b^
Serum glucose (mg/dl)	102.50±6.40	262.50±18.97^a^	114.33±7.01^a^,^b^	105.17±8.02^b^,^c^
Superoxide generation (pmol/min/mg protein)	24.83±6.63	54.17±5.74^a^	23.67±5.37^b^	22.17±8.46^b^
TG (mg/g)	31.00±5.41	131.67±7.16^a^	43.83±9.90^a^,^b^	30.83±5.65^b^,^c^
Hydroxyproline (μmol/mg protein)	2.27±1.51	7.40±1.53^a^	2.93±0.71^b^	2.52±0.86^b^
Serum ALT (U/l)	28.00±4.73	91.67±5.21^a^	36.50±6.40^a^,^b^	27.33±4.46^b^,^c^
Serum AST (U/l)	74.00±6.35	154.50±12.63^a^	78.00±9.81^b^	73.67±9.96^b^
HOMA	2.76±0.49	21.54±2.53^a^	3.47±0.44^b^	3.01±0.60^b^
Body weight (g)	19.50±5.30	23.30±7.10	21.50±6.30	20.60±5.90

Results are expressed as the mean ± standard deviation (n=20 per group).

a P<0.05, vs. control;

b P<0.05, vs. HF;

c P<0.05, vs. HF + heparin.

TG, triglyceride; ALT, alanine transaminase; AST, aspartate aminotransferase; HF, high fat; NASH, nonalcoholic steatohepatitis; HOMA, Homeostatis Model Assessment.
